# 2473. Rates of Antibiotic Resistant Infections in Hospitalized Patients with Hematologic and Non-Hematologic Cancers, 2019

**DOI:** 10.1093/ofid/ofad500.2091

**Published:** 2023-11-27

**Authors:** Natalie McCarthy, Joseph D Lutgring, James Baggs, Hannah Wolford, Sujan Reddy

**Affiliations:** CDC, Atlanta, GA; Division of Healthcare Quality Promotion, Centers for Disease Control and Prevention, Atlanta, GA; CDC, Atlanta, GA; CDC, Atlanta, GA; CDC, Atlanta, GA

## Abstract

**Background:**

Patients with cancer have risk factors for developing both hospital-onset (HO) and antibiotic resistant (AR) infections such as high antibiotic use, long lengths of stay, and device utilization. Understanding the epidemiology of AR infections in this population can inform clinical management and prevention strategies. We aimed to describe the rates and proportions of AR infections in hospitalized patients with cancer.

**Methods:**

Using the PINC AI Healthcare Database, we included adult discharges from 2019 among facilities reporting microbiology data. We identified patients with at least one cancer diagnosis from the Healthcare Cost and Utilization Project’s Clinical Classifications Software Refined categories and Elixhauser Comorbidity Software Refined for ICD-10-CM. We calculated age-standardized rates of AR infections among cancer patients per 10,000 discharges, stratified by community-onset (CO: ≤ day 3 of hospitalization) and HO (≥ day 4 of hospitalization), and hematologic and non-hematologic cancer types. Hematologic cancers included lymphoma, leukemia, myeloma and myelodysplastic syndrome. We also examined the proportion of AR infections in patients with cancer among all AR infections in the cohort.

**Results:**

Of >2 million discharges from 277 facilities, 11.4% were patients with cancer. Compared to all patients, cancer patients had higher rates of infection with CO and HO vancomycin resistant Enterococcus (VRE) and carbapenem resistant Enterobacterales (CRE), and HO Enterobacterales with AR phenotypes suggestive of extended-spectrum β-lactamase (ESBL) production (Table 1). Compared to patients with non-hematologic cancer, patients with hematologic cancer had higher rates of all AR infections, except for CO carbapenem resistant *Acinetobacter* spp. (CRAsp) infections (Table 1). For most AR infections, cancer patients made up a greater proportion of all HO AR infections compared to CO AR infections (Table 2).

**Figure:**
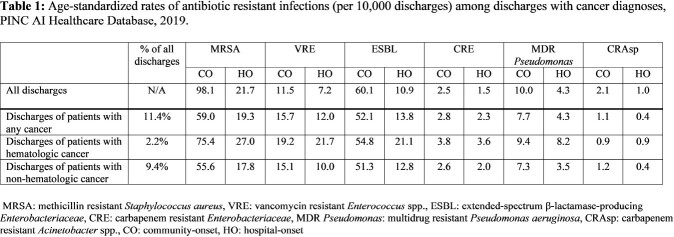

**Figure:**
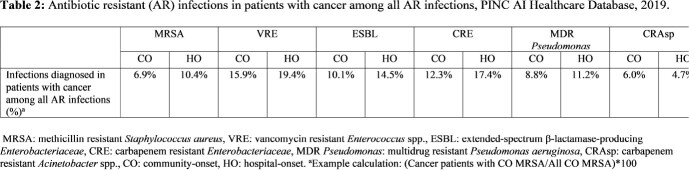

**Conclusion:**

Patients with cancer had higher rates of several AR infections compared to all patients. To decrease the burden of AR infections in this population, infection prevention strategies may focus on VRE, ESBL, and CRE pathogens, as well as HO infections, in all cancer patients, particularly those with hematologic cancer.

**Disclosures:**

**All Authors**: No reported disclosures

